# Immunosuppression as an associated risk factor for high-grade cervical lesions: a comparative study of cytology, HPV, and histopathology

**DOI:** 10.3389/fsurg.2026.1903427

**Published:** 2026-09-15

**Authors:** Merve Baktıroğlu, Mehmet Nuri Duran, Harika Yumru, Hamdullah Sözen, Samet Topuz, Yavuz Salihoğlu

**Affiliations:** 1 Department of Gynecological Oncology, Canakkale Mehmet Akif Ersoy State Hospital, Canakkale, Türkiye; 2 Department of Obstetrics and Gynecology, Ezine State Hospital, Canakkale, Türkiye; 3 Department of Gynecological Oncology, University of Health Sciences, Sisli Hamidiye Etfal Training and Research Hospital, Istanbul, Türkiye; 4 Department of Gynecological Oncology, Faculty of Medicine, Istanbul University, Istanbul, Türkiye

**Keywords:** cervical cytology, cervical intraepithelial neoplasia, high-risk HPV, histopathology, human papillomavirus, immunosuppression

## Abstract

**Background:**

Persistent high-risk human papillomavirus (hrHPV) infection is the main cause of cervical carcinogenesis, and impaired immune surveillance may increase the risk of viral persistence and progression to high-grade lesions. This study aimed to evaluate the association between immunosuppression and high-grade cervical lesions (CIN2+) and to examine its association with HPV status, cytological findings, and histopathological outcomes.

**Methods:**

This retrospective comparative cohort study included women evaluated with cervical cytology, HPV testing, colposcopy, and/or histopathology between 2019 and 2024 at two participating centers. After exclusions, 370 women were analyzed, including 313 immunocompetent controls and 57 immunosuppressed patients. HIV-infected women were excluded. The primary outcome was CIN2 + . Logistic regression analysis was performed to evaluate associations with high-grade cervical disease.

**Results:**

Overall HPV positivity was similar between groups, but immunosuppressed women had a significantly higher prevalence of hrHPV infection. Endocervical curettage (ECC) was performed more frequently in the immunosuppressed group, and ECC positivity was also higher. Final histopathology showed fewer normal findings in immunosuppressed women, with numerically higher rates of CIN2, CIN3, and cancer. In multivariable analysis, immunosuppression remained associated with CIN2 + after adjustment (adjusted OR 1.7, 95% CI 1.0-3.0, *p* = 0.04). HPV16, multiple HPV infection, and HSIL cytology were also associated with CIN2 + in the adjusted model. Exploratory subgroup estimates for renal transplantation and selected immunosuppressive therapies were based on small samples and should be interpreted cautiously.

**Conclusion:**

Immunosuppression was associated with a higher risk of high-grade cervical lesions and a less favorable cervical disease profile, supporting closer surveillance in this population.

## Introduction

Cervical carcinogenesis is now widely understood to be driven primarily by persistent infection with oncogenic human papillomavirus (HPV), rather than by transient exposure alone. Although HPV infection is extremely common in sexually active populations, most infections are cleared spontaneously, indicating that persistence of the virus rather than initial acquisition is the critical step in the development of cervical intraepithelial neoplasia and invasive cancer (1). The ability of the host to eliminate HPV is largely dependent on an effective immune response, particularly cell-mediated immunity involving T lymphocytes and coordinated cytokine signaling. When this immune control is disrupted, viral clearance becomes less efficient, allowing HPV to persist and increasing the probability of progression toward high-grade lesions (2).

Recent research has also highlighted that HPV is not a passive agent but actively interacts with the host immune system. The virus has developed several mechanisms to evade immune detection, including interference with antigen presentation and modulation of local immune signaling pathways. These strategies enable HPV to remain undetected within epithelial tissues and sustain long-term infection (3). The clinical relevance of these mechanisms becomes especially evident in immunocompromised individuals. In such populations, weakened immune surveillance is associated with prolonged viral persistence and a higher likelihood of developing cervical dysplasia and malignancy (4). Immunosuppressed women represent a diverse clinical group, including solid organ transplant recipients, patients with autoimmune diseases requiring long-term immunosuppressive therapy, and those with hematologic conditions. In these settings, chronic immunosuppression affects both innate and adaptive immune pathways, creating conditions that may favor HPV persistence and disease progression (5). Beyond systemic immunity, local immune regulation within the cervical microenvironment also appears to play a meaningful role. Variations in cytokine profiles, alterations in T-cell subsets, and changes in mucosal immune responses have all been linked to both persistence of HPV infection and progression to higher-grade lesions (6). From a virological perspective, certain HPV genotypes, most notably HPV16 and HPV 18 are well known for their strong oncogenic potential. These high-risk types are more likely to persist and to induce neoplastic transformation, particularly when host immune defenses are compromised. In addition, the presence of multiple HPV genotypes and disturbances in the local microenvironment, such as vaginal dysbiosis, may further amplify this risk (7).

Epidemiological data also reinforce this biological framework. Studies have shown that immunosuppressed women tend to have higher rates of persistent HPV infection, increased prevalence of abnormal cytology, and a greater burden of cervical intraepithelial neoplasia compared with immunocompetent populations. This pattern likely reflects not only impaired viral clearance but also prolonged exposure to oncogenic HPV types and a potentially faster progression of premalignant lesions. Furthermore, emerging evidence suggests that disease behavior and screening outcomes in immunosuppressed individuals may differ from those observed in the general population, underscoring the need for more tailored clinical approaches in this group. Despite the growing body of evidence regarding these mechanisms, the overall clinical impact of immunosuppression on cervical disease progression has not been fully clarified. Much of the available literature focuses on specific subgroups, and studies that simultaneously evaluate cytology, HPV status, and histopathological outcomes in a unified framework remain limited (8). In addition, it is still uncertain to what extent immunosuppression is associated to the development of high-grade cervical lesions when other established risk factors such as HPV genotype and smoking are taken into account (9). The aim of this study was to evaluate the association between immunosuppression and high-grade cervical lesions (CIN2+) and to examine whether this association persisted after adjustment for selected clinical, cytological, and HPV-related variables.

## Methods

This study was designed as a retrospective comparative cohort analysis conducted at a tertiary care centers. Medical records of women who underwent cervical cancer screening and/or diagnostic evaluation between January 2019 and December 2024 were systematically reviewed. The eligible population was recruited from women referred to the colposcopy/ gynecologic oncology units of Istanbul University Istanbul Faculty of Medicine, also known as Çapa Hospital, and Canakkale Mehmet Akif Ersoy State Hospital for cervical cancer screening abnormalities and/or diagnostic evaluation. Immunosuppressed and immunocompetent women were selected from the this tertiary colposcopy/gynecologic oncology setting and were evaluated according to the this institutional clinical management protocols. Women with documented HIV infection were excluded because the study focused on non-HIV immunosuppression. Immunocompetent controls were not individually matched to immunosuppressed patients; however, baseline demographic variables, including age, parity, and smoking status, were compared between groups. A total of 405 women were initially identified. Of these, 35 women were excluded: 14 because of missing clinical data, 8 because of incomplete cytology results, 6 because of absence of final histopathological confirmation, and 7 because of other predefined exclusion criteria. Therefore, 370 women had sufficient data for the final analysis, corresponding to 91.4% of the initially identified cohort. These patients were subsequently categorized into two groups based on immune status: an immunocompetent control group (*n* = 313) and an immunosuppressed group (*n* = 57) (Figure 1).

**Figure 1:**
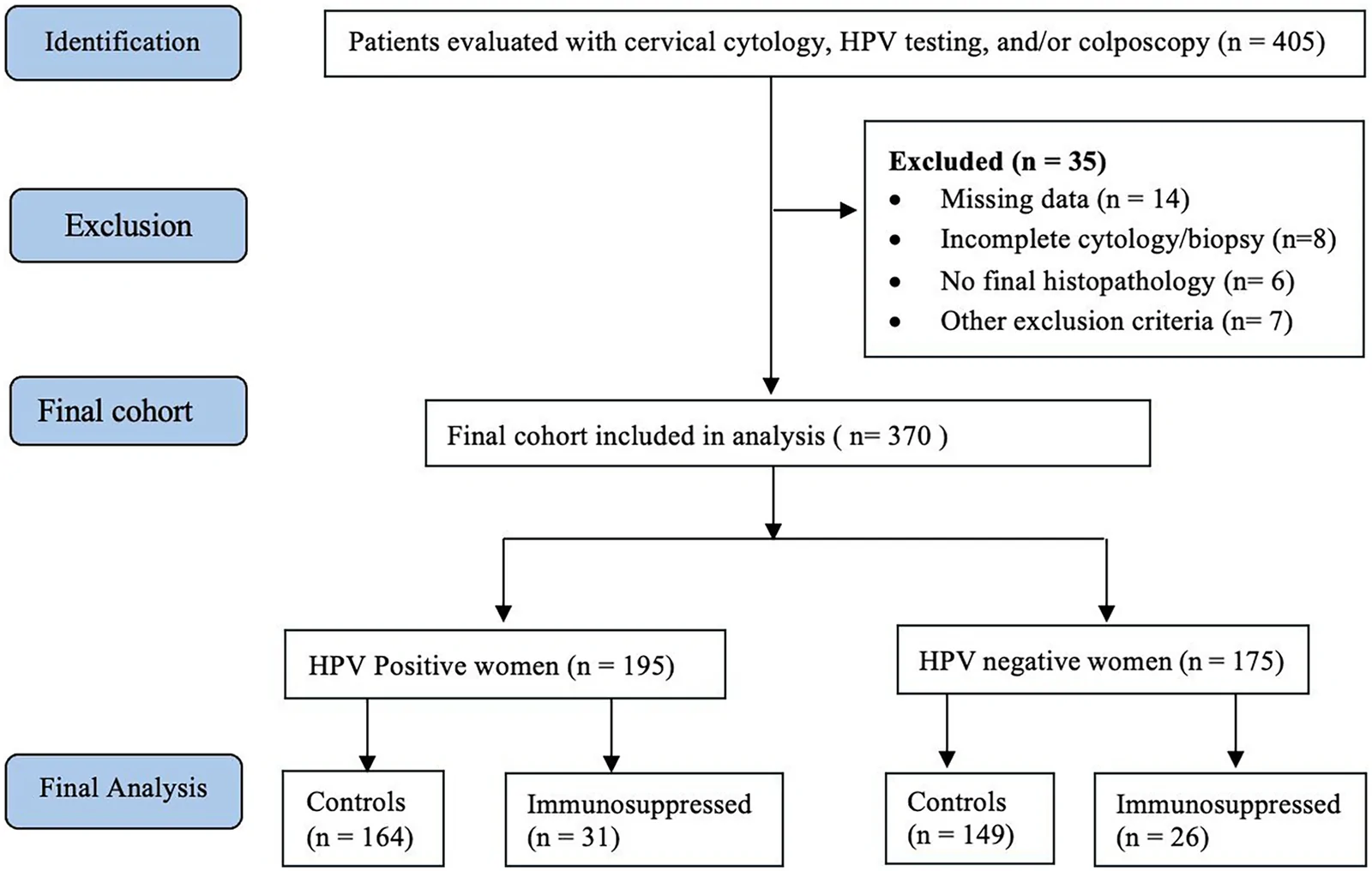
STROBE flow diagram of patient selection and HPV-status distribution in the final cohort.

In the final analytic cohort, HPV test results, cervical cytology, and punch-biopsy findings were available for all 370 women. Endocervical curettage (ECC) was performed in 222 women, including 180/313 immunocompetent controls and 42/57 immunosuppressed patients. Excisional procedures were documented in 32 women according to the available records and were performed according to clinical indications.

Sample collection and laboratory analysis; Cervical samples were obtained during speculum examination using a cervical cytobrush and preserved in a liquid-based cytology medium. HPV DNA detection and genotyping were performed using the F-HPV typing™ multiplex PCR kit (Molgentix SL, Barcelona, Spain), according to the manufacturer's instructions. This assay provides direct type-specific identification of individual HPV genotypes without pooling; therefore, HPV genotypes were recorded separately for each patient when available. High-risk status was assigned according to the type-specific interpretation used by the institutional laboratory protocol. HPV16 and HPV18 were considered separately when detected, and detection of more than one genotype was classified as multiple HPV infection. HPV genotype categories were not mutually exclusive. Immunosuppression was defined as the presence of a chronic condition or treatment known to impair immune function. This included solid organ transplantation, autoimmune diseases requiring long-term immunosuppressive therapy, and hematologic disorders. Solid organ transplant recipients and patients with autoimmune diseases requiring chronic immunosuppressive therapy were therefore included in the immunosuppressed cohort and were not considered exclusion criteria. Pharmacological immunosuppression was further subclassified into corticosteroids, antimetabolites (such as methotrexate, azathioprine, or mycophenolate mofetil), calcineurin inhibitors, biologic agents, and other relevant therapies. Patients with transient or short-term immunosuppression were not included in this group to ensure clinical homogeneity. Detailed information on cumulative treatment duration, drug dose, and overlapping immunosuppressive regimens was not consistently available in the retrospective records and therefore could not be incorporated into the analyses. Demographic and clinical variables were extracted from electronic medical records, including age, parity, smoking status, and underlying comorbidities. HPV testing results were recorded as positive or negative, with additional stratification for high-risk HPV types and genotype distribution when available. Cytological findings were classified according to the Bethesda system, including categories such as ASC-US, LSIL, HSIL, ASC-H, and AGC/AIS. Histopathological outcomes were based on punch biopsy, ECC, and final excisional specimen results, and were categorized as normal, CIN1, CIN2, CIN3, or invasive carcinoma. Histopathological specimens were evaluated by pathologists experienced in gynecologic pathology at the tertiary referral center. Diagnoses were based on routine morphological assessment of punch biopsy, ECC, and excisional specimens. p16 immunohistochemistry was not performed in this cohort. When multiple histopathological specimens were available for the same patient, the final histopathological diagnosis was defined as the most severe lesion identified in any specimen, including punch biopsy, ECC, or excisional specimen. HPV status was defined according to the available HPV test result. Although HPV-negative CIN2 + lesions may reflect false-negative testing, low viral load, sampling limitations, or untested HPV genotypes, these cases were not reclassified as HPV-positive. Because the number of HPV-negative CIN2 + cases was limited, a separate multivariable model for HPV-negative women was not performed.

The primary outcome of interest was the presence of high-grade cervical disease, defined as cervical intraepithelial neoplasia grade 2 or higher (CIN2 +). Secondary outcomes included cytological abnormalities, HPV infection patterns, and concordance between cytology and histopathology.

### Statistical analysis

Statistical analysis was performed using standard statistical software. Continuous variables were expressed as mean ± standard deviation and compared using Student's t-test or Mann–Whitney U test depending on distribution normality. Categorical variables were presented as frequencies and percentages and analyzed using chi-square or Fisher's exact test as appropriate. To evaluate the association between immunosuppression and high-grade cervical lesions after adjustment for selected clinically relevant variables, logistic regression analyses were conducted. Adjusted odds ratios (ORs) with 95% confidence intervals (CIs) were calculated for each covariate. The final multivariable model included immunosuppression status, age, smoking, HPV16 positivity, multiple HPV infection, and HSIL cytology. The primary multivariable model included 370 women. Age was analyzed as a continuous variable. Categorical variables were coded as present vs. absent, with absence used as the reference category. To assess potential detection bias related to differential ECC use, a sensitivity analysis was performed using CIN2 + defined from punch-biopsy findings alone, thereby applying a comparable histopathological assessment across the two immune-status groups.

Covariates were selected *a priori* based on established clinical relevance to cervical neoplasia, with univariate associations used as supportive information rather than as the sole selection criterion. After the predefined exclusions, the primary regression analysis used complete-case data; no imputation was performed. Based on the final histopathological outcome categories, 120 CIN2 + events were available in the 370-woman cohort (93 in immunocompetent women and 27 in immunosuppressed women), corresponding to approximately 20 outcome events per covariate in the six-variable primary model. Model stability was therefore considered primarily in relation to the event-to-covariate ratio and the precision of the reported effect estimates. Formal calibration or discrimination statistics were not retained in the original retrospective statistical output and could not be reliably reconstructed. Disease- and treatment-specific subgroup models were considered exploratory because of the small subgroup sizes, particularly for renal transplant recipients (*n* = 11), calcineurin inhibitor users (*n* = 9), and biologic-agent users (*n* = 6).

## Results

A total of 370 women were analyzed, including 313 immunocompetent and 57 immunosuppressed patients. Baseline characteristics were comparable between groups, with no significant differences in age (43.6 ± 9.4 vs. 44.7 ± 10.1 years, *p* = 0.52), parity (2.1 ± 1.3 vs. 2.2 ± 1.4, *p* = 0.74), or smoking rates (19.8% vs. 14.0%, *p* = 0.30). Overall HPV positivity was similar between the control and immunosuppressed groups (52.4% vs. 54.4%, *p* = 0.48). However, immunosuppressed patients had a higher prevalence of high-risk HPV types, corresponding to an absolute difference of 15.4 percentage points (36.8% vs. 21.4%, *p* = 0.01). Multiple HPV infections were notably less frequent in the immunosuppressed group (10.5% vs. 35.8%, *p* < 0.001). Within the immunosuppressed cohort, autoimmune diseases were the most common underlying condition (66.7%), and corticosteroids were the most frequently used therapy (42.1%), followed by antimetabolites/DMARDs and calcineurin inhibitors (Table 1).

**Table 1:** Baseline demographic, clinical and HPV characteristics.

Variable	Control (*n* = 313)	Immunosuppressed (*n* = 57)	*p*-value
Age (years)	43.6 ± 9.4	44.7 ± 10.1	0.52
Parity	2.1 ± 1.3	2.2 ± 1.4	0.74
Smoking (%)	62 (19.8%)	8 (14.0%)	0.30
HPV status			
HPV positivity	164 (52.4%)	31 (54.4%)	0.48
HPV negative	149 (47.6%)	26 (45.6%)	0.48
High-risk HPV	67 (21.4%)	21 (36.8%)	0.01
hrHPV other than 16/18	40 (12.8%)	3 (5.3%)	0.11
Multiple HPV	112 (35.8%)	6 (10.5%)	<0.001
Comorbidities/reason for immunosuppression			
Renal transplant	-	11 (19.3%)	
Autoimmune diseases	-	38 (66.7%)	
Hematologic/transplant	-	8 (14.0%)	
Immunosuppressive therapy			
Corticosteroids	-	24 (42.1%)	
Antimetabolites/DMARDs	-	18 (31.6%)	
Calcineurin inhibitors	-	9 (15.8%)	
Biologic agents	-	6 (10.5%)	
Colchicine	-	11 (19.3%)	

Values are presented as mean ± standard deviation (SD) for continuous variables and as number (percentage) for categorical variables. Between-group comparisons were performed using Student's t-test or the chi-square test, as appropriate. Multiple HPV infection was defined as detection of more than one genotype. HPV genotype categories were not mutually exclusive; therefore, percentages may exceed 100%. HPV: Human papillomavirus; SD: Standard deviation; DMARDs: Disease-modifying antirheumatic drugs

Cytological findings showed no statistically significant differences between immunocompetent and immunosuppressed women across all categories. Although normal cytology was less frequent in the immunosuppressed group (31.6% vs. 44.7%, *p* = 0.06), this difference did not reach statistical significance. Similarly, the distribution of abnormal cytological findings, including ASC-US, LSIL, HSIL, ASC-H, and AGC/AIS, was comparable between groups. Punch biopsy results followed a similar pattern, with no significant differences observed in the distribution of histopathological diagnoses. Rates of CIN1, CIN2, CIN3, and invasive cancer were numerically higher in the immunosuppressed group; however, these differences were not statistically significant. In contrast, ECC use differed between groups. ECC was performed more frequently in immunosuppressed patients (73.7% vs. 57.5%, *p* = 0.02). When ECC-positive results were expressed as a proportion of the entire immune-status group, the proportions were 35.1% and 22.3%, respectively (*p* = 0.04). Among women who actually underwent ECC, however, positivity was 47.6% (20/42) in immunosuppressed women and 38.9% (70/180) in immunocompetent women (*p* = 0.30). Analysis of final histopathology revealed a lower proportion of normal findings among immunosuppressed women (29.8% vs. 47.9%, *p* = 0.01). Although higher rates of CIN2 and CIN3 were observed in this group, these differences did not reach statistical significance (Table 2).

**Table 2:** Cytological and histopathological findings.

Variable	Control (*n* = 313)	Immunosuppressed (*n* = 57)	*p*-value
Cytology			
Normal	140 (44.7%)	18 (31.6%)	0.06
ASC-US	55 (17.5%)	11 (19.3%)	0.72
LSIL	62 (19.8%)	14 (24.6%)	0.38
HSIL	36 (11.5%)	10 (17.5%)	0.18
ASC-H	12 (3.8%)	3 (5.3%)	0.62
AGC/AIS	8 (2.5%)	1 (1.8%)	0.75
Punch biopsy			
Normal	120 (38.3%)	16 (28.1%)	0.12
CIN1	85 (27.1%)	14 (24.6%)	0.68
CIN2	60 (19.2%)	15 (26.3%)	0.19
CIN3	38 (12.1%)	10 (17.5%)	0.25
Cancer	10 (3.2%)	2 (3.5%)	0.91
ECC			
Performed	180 (57.5%)	42 (73.7%)	0.02
Positive entire group	70 (22.3%)	20 (35.1%)	0.04
Positive among women undergoing ECC	70/180 (38.9%)	20/42 (47.6%)	0.30
Final pathology			
Normal	150 (47.9%)	17 (29.8%)	0.01
CIN1	70 (22.3%)	13 (22.8%)	0.94
CIN2	55 (17.6%)	14 (24.6%)	0.18
CIN3	30 (9.6%)	10 (17.5%)	0.07
Cancer	8 (2.6%)	3 (5.3%)	0.22

Values are presented as *n* (%). *p*-values were obtained using chi-square or Fisher's exact test, as appropriate. For “Positive, entire group,” percentages use the full immune-status group as the denominator; “Positive among women undergoing ECC” uses only women who underwent ECC. ASC-US: Atypical squamous cells of undetermined significance; ECC: Endocervical curettage.

In the sensitivity analysis based exclusively on punch-biopsy findings, CIN2 + was present in 108/313 (34.5%) immunocompetent women and 27/57 (47.4%) immunosuppressed women (crude OR 1.71, 95% CI 0.97-3.02; *p* = 0.07). The direction of the association was similar to the main analysis, although the estimate did not reach statistical significance. In the primary based on the final histopathological outcome categories, immunosuppression was associated with CIN2 + in unadjusted analysis (crude OR 2.1, 95% CI 1.2–3.8; *p* = 0.01), and the association remained statistically significant after adjustment for selected clinically relevant variables (adjusted OR 1.7, 95% CI 1.0–3.0; *p* = 0.04). Age and smoking were not significantly associated with CIN2 + in either unadjusted or adjusted models. Among HPV-related variables, HPV16 infection was associated with high-grade disease (adjusted OR 2.3, 95% CI 1.3–4.1; *p* = 0.003). Multiple HPV infection was also associated with CIN2+ (adjusted OR 1.8, 95% CI 1.0–3.1; *p* = 0.04). HSIL cytology showed the strongest association in the adjusted model (adjusted OR 4.2, 95% CI 2.1–8.3; *p* < 0.001) (Table 3).

**Table 3:** Multivariable logistic regression analysis of risk factors for high-grade cervical lesions (CIN2+).

Variable	Crude OR	95% CI	*p*-value	Adjusted OR	95% CI	*p*-value
Immunosuppressed	2.1	1.2–3.8	0.01	1.7	1.0–3.0	0.04
Age (per year)	1.01	0.98–1.03	0.42	1.00	0.97–1.03	0.88
Smoking	1.4	0.9–2.2	0.11	1.2	0.7–2.0	0.31
HPV16	2.8	1.6–4.9	<0.001	2.3	1.3–4.1	0.003
Multiple HPV	2.1	1.2–3.6	0.006	1.8	1.0–3.1	0.04
HSIL cytology	5.6	3.0–10.4	<0.001	4.2	2.1–8.3	<0.001
Subgroup analysis						
Renal transplant (*n* = 11)	2.5	1.3–4.8	0.005	2.1	1.1–4.1	0.02
Autoimmune disease	1.6	0.9–2.8	0.09	1.4	0.8–2.5	0.15
Hematologic/other	1.8	0.8–3.9	0.12	1.5	0.7–3.3	0.21
Immunosuppressive therapy						
Corticosteroids	2.2	1.2–3.9	0.01	1.9	1.0–3.4	0.04
Antimetabolites (MTX/AZA/MMF)	1.8	1.0–3.2	0.04	1.6	0.9–2.9	0.08
Calcineurin inhibitors (*n* = 9)	2.6	1.2–5.5	0.01	2.2	1.0–4.8	0.04
Biologic agents (*n* = 6)	2.0	0.9–4.3	0.07	1.7	0.8–3.8	0.11

In exploratory subgroup analyses, renal transplant recipients showed a higher CIN2 + estimate, but this result was based on only 11 patients and may be unstable. Similarly, treatment-specific estimates, particularly for calcineurin inhibitor users (*n* = 9) and biologic-agent users (*n* = 6), were based on small samples. These subgroup results are therefore presented as hypothesis-generating findings and should not be interpreted as definitive disease- or treatment-specific effects.

ORs with 95% CIs were calculated using logistic regression. *p* < 0.05 was considered statistically significant. OR: Odds ratio; CI: Confidence interval; CIN: Cervical intraepithelial neoplasia; HSIL: High-grade squamous intraepithelial lesion. The primary multivariable model included 370 women and six covariates: immunosuppression status, age, smoking, HPV16 positivity, multiple HPV infection, and HSIL cytology. Age was continuous. Categorical variables were coded as present vs. absent, with absence as the reference category Disease- and treatment-specific subgroup variables were evaluated in separate exploratory models because of small subgroup sizes. Estimates for renal transplant recipients (*n* = 11), calcineurin inhibitor users (*n* = 9), and biologic-agent users (*n* = 6) may be unstable and should therefore be interpreted cautiously.

Cytological distribution differed modestly between groups. The proportion of normal cytology was lower in immunosuppressed women, whereas abnormal findings, particularly LSIL and HSIL, were relatively more frequent. ASC-US rates were comparable between groups, and no marked differences were observed for less common categories such as ASC-H and AGC/AIS. Overall, group differences in cytological distribution were assessed using the chi-square test, with the null hypothesis that cytological categories were similarly distributed between groups. Although this comparison did not reach statistical significance, the pattern suggests a shift toward higher-grade cytological abnormalities in the immunosuppressed population (Figure 2).

**Figure 2:**
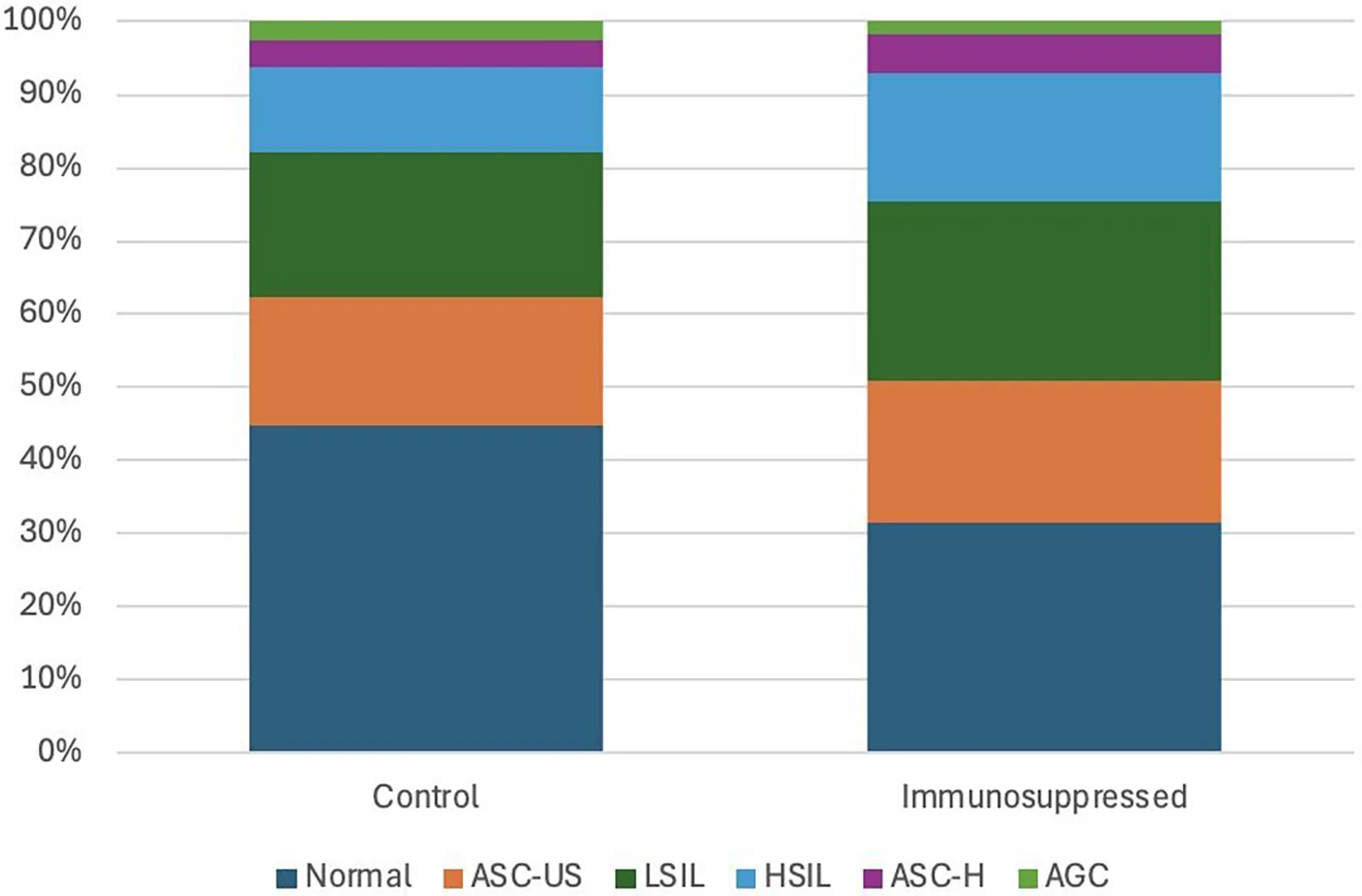
Distribution of cytological findings between control and immunosuppressed groups.

Bars represent the percentage distribution of cytological categories within each group. Exact values and statistical comparisons are provided in Table 2. ASC-US: Atypical squamous cells of undetermined significance; LSIL: Low-grade squamous intraepithelial lesion; HSIL: High-grade squamous intraepithelial lesion; ASC-H: Atypical squamous cells cannot exclude HSIL; AGC/AIS: Atypical glandular cells/adenocarcinoma *in situ*.

Directed acyclic graph illustrating the assumed causal structure between immunosuppression, HPV acquisition or persistence, HPV-related biological context, and CIN2 + (Figure 3). HPV-related variables were represented as part of the biological pathway rather than as simple confounders. The referral and sampling pathway was included to reflect potential detection bias related to ECC and biopsy use. The diagram was used to guide interpretation of the adjusted analyses and does not establish causality.

**Figure 3:**
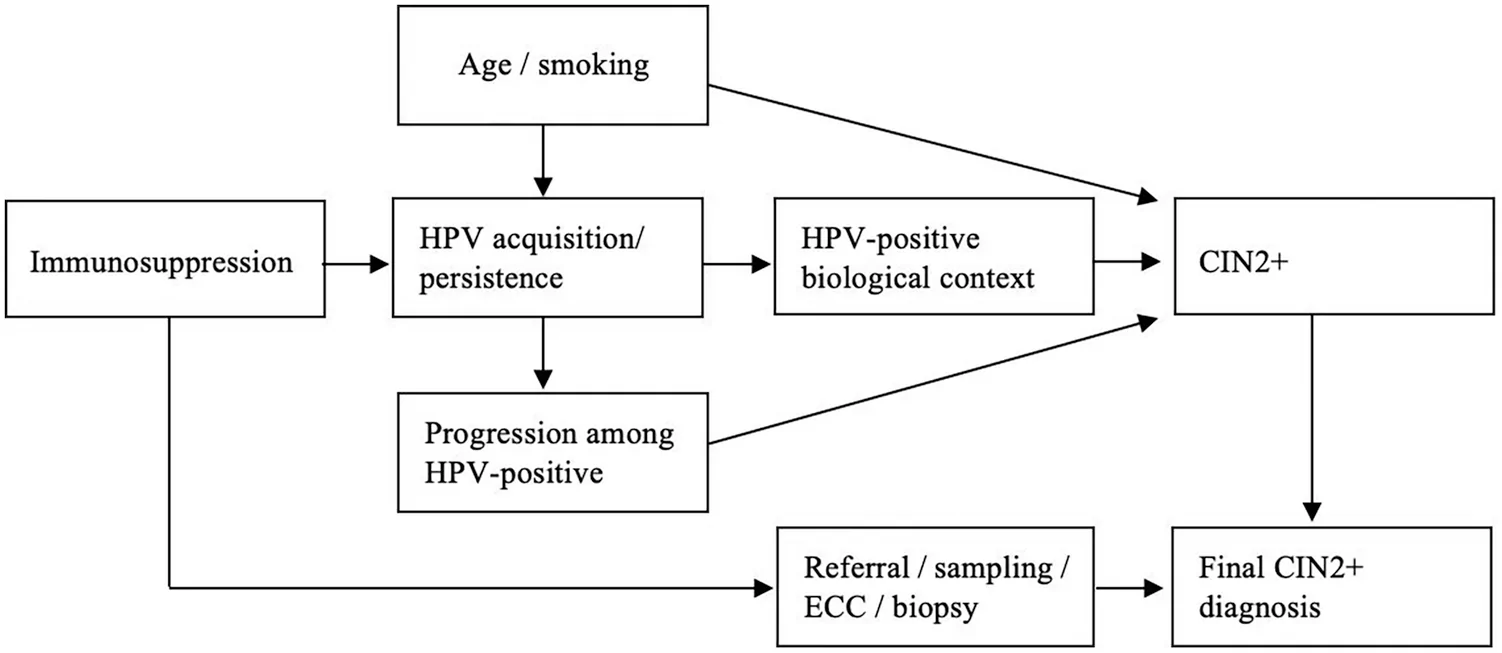
Directed acyclic graph of the assumed causal pathway between immunosuppression, HPV status, and CIN2+.

## Discussion

In the present study, immunosuppression remained associated with CIN2 + after adjustment for established risk factors, including age, smoking, HPV16 positivity, multiple HPV infection, and HSIL cytology. Rather than reflecting only a higher overall HPV positivity rate, immunosuppressed women demonstrated a distinct cervical disease profile characterized by higher hrHPV prevalence and less favorable histopathological outcomes. This finding is clinically important because it suggests that immune status may contribute to cervical neoplastic progression beyond conventional risk markers alone. Our results are broadly consistent with recent large-scale literature showing that chronic systemic immunosuppression facilitates persistent HPV infection, impairs viral clearance, and increases the likelihood of progression to cervical precancer. In an umbrella review, systemic immunosuppression and immunosuppressive medications were among the most consistently supported risk factors for worse HPV- and cervical disease-related outcomes (10). Although the overall HPV positivity rate in our cohort was not significantly different between groups, the immunosuppressed group showed a distinctly different virologic profile, particularly with a clinically meaningful absolute difference in the prevalence of high-risk HPV. An unexpected finding was the lower frequency of multiple HPV infections in immunosuppressed women despite a higher prevalence of high-risk HPV. Because HPV genotyping was performed using the same type-specific multiplex PCR assay and the same institutional laboratory protocol in both groups, this difference is unlikely to be explained by differential assay sensitivity. Nevertheless, this finding should be interpreted cautiously. It may reflect the relatively small size of the immunosuppressed group, referral-related selection bias, differences in underlying clinical characteristics, or the predominance of single high-risk genotypes in some immunosuppressed patients. Detailed data on sexual behavior were not available in this retrospective dataset; therefore, differences in sexual exposure patterns between groups cannot be excluded as a possible contributing factor. Accordingly, this observation should be considered hypothesis-generating rather than definitive. This pattern may be more informative than crude HPV positivity alone, because in immunosuppressed patients the clinically relevant issue is not merely infection, but the persistence of oncogenic genotypes and their progression potential. In female renal transplant recipients, Chen et al. reported increased hrHPV prevalence and a higher risk of CIN2 + compared with controls, with HPV16 being especially overrepresented. A 2024 systematic review and meta-analysis by Ring et al. similarly showed that kidney transplant recipients had a pooled cervical hrHPV prevalence of 27.7%, and that hrHPV prevalence was significantly higher than in immunocompetent controls (11, 12). Recent transplant-focused studies consistently indicate that transplant recipients represent one of the highest-risk non-HIV immunosuppressed populations for HPV-related lower genital tract disease. In a 2025 cross-sectional study, Hillen et al. found that female transplant recipients had a cervical hrHPV prevalence of 15.9%, with rates above those expected in the general population, particularly in women older than 30 years (12, 13). Given the small renal transplant subgroup (*n* = 11), the transplant-specific estimate should be considered exploratory and hypothesis-generating rather than a stable subgroup effect.

By contrast, the autoimmune disease subgroup in our study showed only a numerical increase that did not remain statistically significant after adjustment. This more modest effect is also consistent with recent evidence suggesting that autoimmune populations are heterogeneous, and that risk varies substantially according to the underlying disease and the intensity of immunosuppressive exposure. Foster et al., using a large Australian linkage cohort, showed that women with autoimmune conditions had increased low-grade cervical abnormalities overall, while high-grade abnormalities were particularly more pronounced in SLE/MCTD rather than uniformly across all autoimmune diagnoses (14). More recently, Lee et al. reported that women of childbearing age with rheumatic diseases had an approximately threefold higher risk of HPV-associated gynecologic cancers overall, but the magnitude differed across subgroups, being highest in seropositive rheumatoid arthritis and still elevated in SLE. This pattern fits with the broader concept that both the degree and duration of immunosuppression matter. In women with inflammatory bowel disease, Goetgebuer et al. demonstrated an increased risk of high-grade cervical neoplasia, and Kreijne et al. later showed that cumulative exposure to immunomodulators further increased cervical neoplasia risk (15). The immunosuppressed cohort was clinically heterogeneous, including patients with different underlying diseases and receiving different classes of immunosuppressive therapy. This heterogeneity may have influenced the magnitude of the observed associations and limits disease- and treatment-specific interpretation. Similarly, recent reviews of autoimmune and transplant populations suggest that prolonged exposure to agents such as corticosteroids, azathioprine, tacrolimus, cyclosporine, and mycophenolate may be more relevant than diagnosis alone in shaping HPV persistence and neoplastic risk (16). Cytologically, immunosuppressed women had numerically higher rates of HSIL, ASC-H, and LSIL, although not all comparisons reached statistical significance, likely due to the relatively limited sample size of this subgroup. However, HSIL cytology remained one of the strongest predictors in the adjusted modelof CIN2 + in our regression model, which is entirely consistent with real-world colposcopy data. Histopathologically, immunosuppressed women also had fewer normal final pathology results and more CIN2, CIN3, and cancer, supporting the interpretation that immunosuppression is associated not only with abnormal screening findings but also with a more clinically meaningful disease spectrum. We also observed significantly higher ECC performance and ECC positivity rates in the immunosuppressed group. Although ECC specific data in non-HIV immunosuppressed populations remain limited, this finding may suggest a greater likelihood of endocervical or occult disease that is not fully captured by punch biopsy alone. Recent colposcopy literature continues to support the diagnostic contribution of ECC in higher-risk settings, particularly in women with high-grade cytology or other concerning findings (17). HPV-negative high-grade lesions are generally considered uncommon and may reflect sampling limitations, assay-related factors, low viral load, or post-transformational viral clearance rather than a truly HPV-independent mechanism (18). Treatment-specific estimates were also exploratory. In particular, the calcineurin inhibitor subgroup included only nine patients, and the biologic-agent subgroup included six; these estimates may therefore be unstable and should not be interpreted as definitive treatment effects.

Importantly, when CIN2 + was defined using punch-biopsy findings alone, the association remained directionally similar but was attenuated and no longer statistically significant. This finding suggests that differential diagnostic intensity, particularly the more frequent use of ECC in immunosuppressed women, may have influenced the magnitude and precision of the observed association and supports cautious interpretation. Overall, our findings indicate that immunosuppression is associated with a clinically more consequential cervical disease profile characterized by greater high-risk HPV enrichment, higher ECC positivity, fewer normal final pathology results, and an increased risk of CIN2 + after adjustment for selected variables. Recent evidence by Preti et al. also emphasizes that screening modality and program organization may influence lesion extent and severity. In a 2026 study, HPV-based detection was associated with CIN3 lesions of smaller linear extension, less extensive glandular crypt involvement, and a lower risk of stromal microinvasion compared with cytology-based detection. In another 2026 study, organized screening was associated with smaller CIN3 lesions, lower cone volumes, and lower invasive cancer rates compared with opportunistic screening. These findings are relevant to our cohort because immunosuppressed women may require not only closer surveillance, but also structured and HPV-based follow-up strategies to detect high-grade lesions at an earlier and potentially less extensive stage (19, 20). These results support closer surveillance and a lower threshold for comprehensive diagnostic evaluation in immunosuppressed women, while disease- and treatment-specific risks should be clarified in larger studies. Future prospective studies with larger subgroup samples are needed to clarify the relative contributions of underlying disease, treatment type, and cumulative immunosuppressive burden. Because HPV-related variables and cytological abnormalities may partly lie on the causal pathway between immunosuppression and CIN2+, the adjusted models should not be interpreted as proving a fully independent causal effect. Rather, they indicate that the association between immunosuppression and CIN2 + persisted after adjustment for selected clinically relevant variables.

### Limitations

This study has limitations. Because the cohort was derived from women referred for colposcopic and/or diagnostic cervical evaluation at a tertiary center, it may be enriched for abnormal screening findings. Therefore, the results may not be directly generalizable to routine population-based screening populations. Concomitant genital infections, including Chlamydia trachomatis, herpesvirus infections, and bacterial vaginosis, were not systematically screened or documented in all patients and therefore could not be included in the analyses. These unmeasured factors may have contributed to residual confounding in the observed associations. Detailed data on sexual behavior were also unavailable, so differences in exposure patterns between groups could not be excluded. In addition, ECC was performed more frequently in immunosuppressed women, which may reflect a lower clinical threshold for more extensive sampling in this higher-risk group. Therefore, higher whole-cohort ECC-positive proportions may partly reflect detection bias rather than only a true difference in disease burden. The lack of standardized information on treatment duration, cumulative dose, and overlapping immunosuppressive therapies limited a more detailed exposure-response assessment. This differential diagnostic intensity may also have contributed to some of the observed between-group differences in final histopathological outcomes. The immunosuppressed cohort was relatively small, and subgroup estimates were particularly limited for renal transplant recipients (*n* = 11), calcineurin inhibitor users (*n* = 9), and biologic-agent users (*n* = 6). These exploratory estimates may be unstable and require confirmation in larger cohorts. Formal calibration and discrimination diagnostics were not available from the archived original statistical output, which limits further assessment of model performance.

## Conclusion

In conclusion, our findings indicate that immunosuppression is associated with an increased risk of high-grade cervical lesions. Although overall HPV positivity was similar between groups, immunosuppressed women demonstrated a less favorable cervical disease profile, including a higher prevalence of high-risk HPV, increased ECC positivity, and fewer normal final histopathological findings. In addition, HPV16 positivity, multiple HPV infection, and HSIL cytology remained strong predictors of CIN2 + . These results suggest that immunosuppressed women may require closer surveillance and a lower threshold for comprehensive diagnostic evaluation during cervical cancer screening and follow-up. Larger prospective studies are needed to clarify the relative impact of different causes of immunosuppression and treatment-related factors on cervical disease progression. Signals observed in disease- and treatment-specific subgroup analyses should be regarded as exploratory because of the small subgroup sizes and require validation in larger studies.

## Data Availability

The raw data supporting the conclusions of this article will be made available by the authors, without undue reservation.
